# Machine Learning-Based Prediction Models for Cognitive Decline Progression: A Comparative Study in Multilingual Settings Using Speech Analysis

**DOI:** 10.14283/jarlife.2024.6

**Published:** 2024-05-16

**Authors:** B. Ceyhan, S. Bek, T. Önal-Süzek

**Affiliations:** 1 Department of Bioinformatics, Graduate School of Natural and Applied Sciences, Mugla Sitki Kocman University, Mugla 48000, Türkiye; 2 Department of Neurology, Faculty of Medicine, Mugla Sitki Kocman University, Mugla 48000, Türkiye

**Keywords:** Spontaneous speech, machine learning, dementia, mild cognitive impairment, mental status and dementia tests

## Abstract

**Background:**

Mild cognitive impairment (MCI) is a condition commonly associated with dementia. Therefore, early prediction of progression from MCI to dementia is essential for preventing or alleviating cognitive decline. Given that dementia affects cognitive functions like language and speech, detecting disease progression through speech analysis can provide a cost-effective solution for patients and caregivers.

**Design-Participants:**

In our study, we examined spontaneous speech (SS) and written Mini Mental Status Examination (MMSE) scores from a 60-patient dataset obtained from the Mugla University Dementia Outpatient Clinic (MUDC) and a 153-patient dataset from the Alzheimer’s Dementia Recognition through Spontaneous Speech (ADRess) challenge. Our study, for the first time, analyzed the impact of audio features extracted from SS in distinguishing between different degrees of cognitive impairment using both an Indo-European language and a Turkic language, which exhibit distinct word order, agglutination, noun cases, and grammatical markers.

**Results:**

When each machine learning model was tested on its respective trained language, we attained a 95% accuracy using the random forest classifier on the ADRess dataset and a 94% accuracy on the MUDC dataset employing the multilayer perceptron (MLP) neural network algorithm. In our second experiment, we evaluated the effectiveness of each language-specific machine learning model on the dataset of the other language. We achieved accuracies of 72% for English and 76% for Turkish, respectively.

**Conclusion:**

These findings underscore the cross-language potential of audio features for automated tracking of cognitive impairment progression in MCI patients, offering a convenient and cost-effective option for clinicians or patients.

## Introduction

**E**arly detection of cognitive impairment on a population scale would benefit both individuals and society, including improved quality of life for affected individuals, decreased healthcare costs linked to late-stage treatment, and the chance for targeted resource allocation in healthcare systems. Nevertheless, existing detection techniques in clinics tend to be intrusive or lengthy, making them impractical for the ongoing observation of asymptomatic individuals. For instance, gathering biological indicators of neuropathology linked to cognitive decline usually requires cerebral spinal fluid samples, while cognitive performance is assessed through in-person evaluations by specialists, and brain metrics are obtained using costly, immobile equipment. Presently, the global population of individuals afflicted with dementia exceeds 55 million, with 60-70% of these cases attributed to Alzheimer’s disease, rendering it the predominant form of dementia (1). It stands as a primary contributor to dependency among older individuals, presenting caregivers with formidable challenges due to decreased physical engagement and mood alterations. Hence, it holds great importance to vigilantly monitor the progress of individuals aged 65 years and older, particularly those exhibiting no discernible symptoms or presenting with mild cognitive impairment (MCI), with the aim of forestalling or mitigating cognitive deterioration (2).

Several clinical tools and imaging techniques help estimate the course of dementia. For example, the Cardiovascular Risk Factors, Aging, and Incidence of Dementia (CAIDE) Risk Score was designed to predict the risk of developing dementia within 20 years for middle-aged people. The Brief Dementia Screening Indicator (BDSI) aims to identify older patients to target for cognitive screening by determining their risk of developing dementia within 6 years (2). The MMSE is a common screening tool for dementia, and it is primarily used by clinicians to assess cognitive decline. However, these tests are generally performed in clinical environments, and patients do not take them unless there are symptoms or avoid repeating them due to an unwillingness to visit these institutions.

Considering the limited accessibility, older patients’ reluctance to undergo standard laboratory cognitive tests, and the urgent need to prevent Mild Cognitive Impairment (MCI) from progressing to advanced stages, nonclinical and non-drug-based tools garner increased attention for thorough investigation. Advances in smartphone technology facilitate effortless passive monitoring of speech, fine motor skills, and gait patterns. Despite several challenges, such as cross-cultural adaptation (4) and standardization associated with these home-based prediction systems, they have the potential to assist in predicting cognitive decline at an earlier stage. Patients with MCI and dementia are known to have language difficulties such as word finding, sentence comprehension in producing speech, acoustic parameters such as shimmer, and number of voice breaks that significantly differentiate them from healthy adults (5). A study that conducted machine learning (ML) modeling by extracting linguistic features at the syntactic, semantic, and pragmatic levels from patient speech data achieved 79% accuracy in distinguishing Alzheimer’s disease patients from healthy adults using support vector machines (SVMs), neural networks (NNs) and decision tree classifiers (6). For acoustic feature research, another study used the Dementia Bank dataset, and 94.71% accuracy was achieved using the Bayes Net classification on 263 features of the audio files (7). For a non-English speaking study, a Spanish study used machine learning to extract linguistic features from spontaneous speech to detect cognitive impairments and achieved accuracies between 65% and 80% (8).

In this study we used the Alzheimer’s Dementia Recognition through Spontaneous Speech (ADReSS) dataset obtained from the ADRess challenge (11) and a Turkish patient dataset collected from the Mugla University Dementia Clinic (MUDC) when creating two ML models for evaluating the accuracy of speech-based acoustic features for predicting the MMSE score of patients. The ADRess dataset has 153 audio recordings of English-speaking older adults, while the MUDC dataset has 60 Turkish audio recordings of dementia clinic patients collected via the application and a website created for this purpose. Both datasets are composed of cognitively normal (CN) and AD patients balanced in terms of age and sex along with their written MMSE scores. This study preferred acoustic feature-based modeling because MMSE prediction is applied to Turkish-speaking patients. Linguistic modeling is affected by language and culture, and the automation of linguistic extraction features has not been successful in several studies (10).

## Methods

### Dataset

Two audio datasets, one obtained from the ADReSS dataset and one from 60 participants in the MUDC, were used for MMSE prediction via speech ML modeling. The breakdown of patients in the ADReSS dataset is given in Table 1; it is a balanced dataset composed of cognitively normal (CN) patients and those suffering from Alzheimer’s disease (AD) who were requested to talk about Cookie theft pictures used in the Boston Diagnostic Aphasia Exam (BDAE) (11). In addition to the SS recordings, the Pitt corpus of the ADReSS contained the written MMSE scores, age, and gender of all participants. The Pitt corpus dataset was obtained from the ADReSS challenge website by becoming a member of the Dementia Data Bank (12).

**Table 1.** Breakdown of ADReSS Training Dataset participants by Gender and AD status

	AD		non-AD	
Age Interval	Male	Female	Male	Female
[50, 55)	2	0	2	0
[55, 60)	7	6	7	6
[60, 65)	4	9	4	9
[65, 70)	9	14	9	14
[70, 75)	9	11	9	11
[75, 80)	4	3	4	3
Total	35	43	35	43

To compare the performance of both datasets and assess whether the underlying language’s linguistic features were critical factors in this assessment, we repeated the same Cookie Theft picture exam in Turkish on 60 patients with mild cognitive impairment in the MUDC. The MUDC performed the written MMSE tests on all these patients within one month before the SS recordings were retrieved. Table 2 summarizes the participants’ metadata.

**Table 2.** Breakdown of MUDC Dataset participants by Gender and MMSE Scores

n=60		Age Range (< 60)	Age Range (60-69)	Age Range (> 70)
Female	MMSE > 25	2	7	7
Female	MMSE 20-25	0	3	7
Female	MMSE < 20	2	3	4
Total	35			
Male	MMSE > 25	2	4	1
Male	MMSE 20-25	0	4	8
Male	MMSE < 20	1	2	3
Total	25			

### Audio Data Collection and Preprocessing

**Figure 1.**
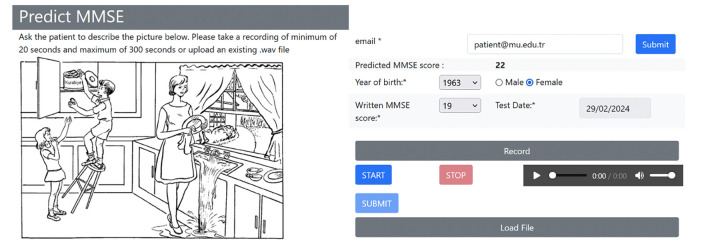
Patient recording and MMSE prediction page

**Figure 2.**
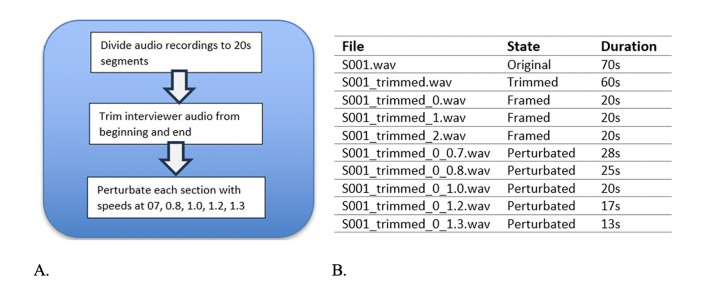
(A) Audio file augmentation process steps. (B) Perturbation step from one file to 15 files

A total of 153 audio recordings in the ADReSS dataset and 60 in the MUDC dataset were separately preprocessed and analyzed because both recordings were recorded in different environments with different languages. While the ADReSS dataset was obtained from the Dementia Bank website (13), Figure 2 shows the website developed for this study to capture recordings and check MMSE predictions for the MUDC dataset. A clinician conducted the assessments in Turkish within a calm and controlled environment. Immediately after their formal MMSE cognitive test at the clinic, each participant was presented with the Cookie Theft Picture and was instructed to provide a comprehensive description of the image within 1 minute (Figure 1). The participants’ voices were recorded during the test administration, and the collected data were utilized for further analyses.

The preprocessing of the audio data involved framing, trimming, and augmenting the audio files programmatically to enable repeating this process in both datasets. One of the main purposes of preprocessing was to increase the sample size for each dataset to avoid overfitting and reduce bias. The first step in augmentation was to divide the audio files into smaller but more stationary ones. The average duration of the audio files in the ADReSS dataset is 80 seconds, so we performed both manual and programmatic analyses to determine the optimal segment duration. For the latter, we developed code to go over audio files to find the average lowest total harmonic distortion, a measure that represents the distortion rate in a segment divided by the total distortion in the file (14), which was 15 seconds. After the recordings, the files were programmatically framed into 20-second segments using Python Librosa libraries to have enough words in each segment for a sentence (Figure 2A). After we trimmed the interviewer audio from the beginning and end using a 5-second buffer, we programmatically perturbed each section at speeds of 0.7, 0.8, 1.2, and 1.3 to create 1520 samples from 153 samples for the training dataset and 800 samples from 48 samples for the test dataset (Figure 2B). The same process was applied to the MUDC dataset, and 60 samples were augmented to 300.

### Feature Extraction and ML Model Creation from Audio Files

**Figure 3.**
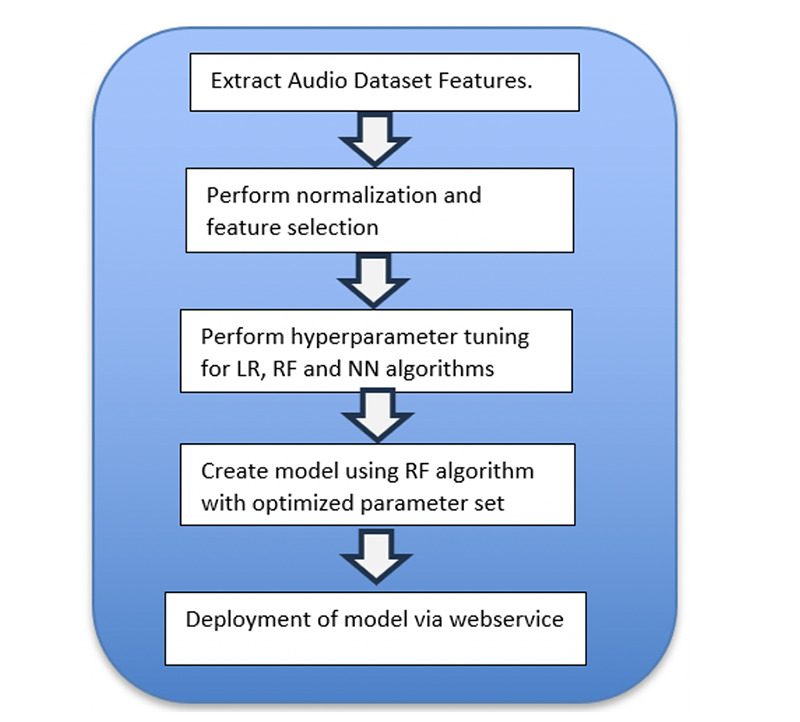
Audio file feature extraction and ML model creation steps

Figure 3 displays the steps we followed after the audio files were preprocessed and augmented. The resulting model is deployed to the application server as a web service called by the website in Figure 2.

**Figure 4.**
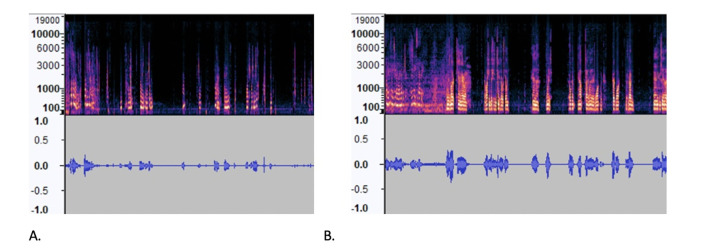
Waveform and Spectrogram of (A) AD recording (B) CN recording

The spectrograms in MEL format and waveforms in Figure 4 clearly show differences in the number of peaks, energy levels and pauses between the AD and CN audio files. To measure the differences between these two samples, Table 3 lists extracted audio features such as the root mean square (RMS), zero crossing rate (ZCR), spectrum features, number of silence of segment, skewness and mean, and standard deviation of 30 mel-frequency cepstral coefficients (MFCC), which have been used in other studies that analyzed the ADRess dataset (7, 13). MFCCs are one of the most popular feature extraction techniques used in speech recognition based on frequency domain using the Mel scale which is based on the human ear scale (16). Due to the high variation in MFCC signals, we included the mean and standard variation of this measure. Feature selection using the KBest algorithm did not eliminate any of these features, as the average accuracy rate of the model using 10-fold cross-validation was lower with fewer selected features in each case.

**Table 3.** Audio features extracted from datasets

Feature	Description
Duration	Duration of audio in seconds
Root Mean Square (RMS)	measure of the amplitude or loudness of an audio signal
Zero Crossing Rate (ZCR)	the rate at which a signal changes its sign, crossing the zero-amplitude axis
Spectral Centroid	the average frequency of a signal
Spectral Bandwidth	the width or spread of the frequency content in a signal’s spectrum
Spectral Contrast	the difference in magnitude between peaks and valleys in the spectrum of a signal
Mel-Frequency Cepstral Coefficients (MFCC)	a measure that captures the characteristics of the human voice. Mean and standard variation of 30 captures were extracted
Skewness	a measure of the asymmetry of the spectrum around the spectral centroid. Mean of skewness of 30 captures were extracted

After preparing and preprocessing the audio data and extracting the audio features, we performed GridSearchCV hyperparameter optimization using 10-fold cross-validation for logistic regression (LR), random forest (RF) and neural network (NN)-multilayer perceptron (MLP) algorithms for both datasets. Table 4 shows the hyperparameter tuning results, best hyperparameters, means and standard deviations between each set of fold results with and without normalization and feature selection.

**Table 4.** ADReSS dataset hyperparameter optimization results

Algorithm	Optimized Parameters and Accuracy Rates
Data not augmented– Feature selection NOT performed - 98 Features	
LR	Best hyperparameters: {‘C’: 10, ‘penalty’: ‘l2’} Mean cross-validation score: 0.2627
RF	Best hyperparameters: {‘max_depth’: 100, ‘max_features’: ‘sqrt’, ‘n_estimators’: 500} Mean cross-validation score: 0.5232 SD cross-validation score: 0.0900
NN	Best hyperparameters: {‘alpha’: 0.001, ‘hidden_layer_sizes’: (100,)} Mean cross-validation score: 0.3095 SD cross-validation score: 0.0776
Data not normalized – Feature selection NOT performed - 98 Features	
LR	Best hyperparameters: {‘C’: 1, ‘penalty’: ‘l2’} Mean cross-validation score: 0.7919
RF	Best hyperparameters: {‘max_depth’: 100, ‘max_features’: sqrt, ‘n_estimators’: 500} Mean cross-validation score: 0.8501
NN	Best hyperparameters: {‘alpha’: 0.01, ‘hidden_layer_sizes’: (100,)} Mean cross-validation score: 0.8603
Data normalized – Feature reduction performed – 30 features	
LR	Best hyperparameters: {‘C’: 10, ‘penalty’: ‘l2’} Mean cross-validation score: 0.5941
RF	Best hyperparameters: {‘max_depth’: None, ‘max_features’: ‘sqrt’, ‘n_estimators’: 500} Mean cross-validation score: 0.9586 SD cross-validation score: 0.0570
NN	Best hyperparameters: {‘alpha’: 0.01, ‘hidden_layer_sizes’: (100,)} Mean cross-validation score: 0.9237 SD cross-validation score: 0.0914

## Results

### Acoustic Analysis

For the ADReSS dataset, the best classification algorithm was the random forest (RF) algorithm, which achieved 95.79% accuracy with normalization and feature reduction. For feature normalization, the Python StandardScaler function was used. Table 5 displays the average accuracy, F1, and AUC scores when using a 10-fold cross-validation score for the RF algorithm, which resulted in the best accuracy of 95%. The difference in the mean cross-validation score between the augmented and unaugmented data clearly demonstrates the value of increasing the sample size for model creation.

To perform independent dataset validation with a 70-30 train-test split, we implemented validation with the Adress Test Dataset, which had 60 recordings with a balanced population of gender and MMSE scores. We achieved 73% accuracy for the labels (dementia or not) and a root mean square error (RMSE) of 5.6 for the MMSE predictions (Table 4, 5).

**Table 5.** ADReSS dataset Classification Accuracy Scores

**Classifier**	**Accuracy**
LR	56%
RF	95%
NN	92%
RF	73% using Test Dataset, RMSE 5.6 on MMSE Predictions
**Random Forest**	**Classifier results**
Accuracy	95% (+/- 0.07)
F1 Weighted	95% (+/- 0.07)
Mean AUC	94%

**Table 6.** MUDC dataset hyperparameter optimization results

Algorithm	Optimized Parameters and Accuracy Rates
Data not normalized – Feature selection NOT performed - 98 Features	
LR	Best hyperparameters: {‘C’: 0.1, ‘penalty’: ‘l2’} Mean cross-validation score: 0.4359
RF	Best hyperparameters: {‘max_depth’: 100, ‘max_features’: ‘log2’, ‘n_estimators’: 100} Mean cross-validation score: 0.6571 SD cross-validation score: 0.0763
NN	Best hyperparameters: {‘alpha’: 0.01, ‘hidden_layer_sizes’: (100,)} Mean cross-validation score: 0.6355 SD cross-validation score: 0.1518
Data normalized – Feature reduction performed – 30 features	
LR	Best hyperparameters: {‘C’: 100, ‘penalty’: ‘l2’} Mean cross-validation score: 0.8859
RF	Best hyperparameters: {‘max_depth’: None, ‘max_features’: ‘sqrt’, ‘n_estimators’: 100} Mean cross-validation score: 0.9193 SD cross-validation score: 0.1051
NN	Best hyperparameters: {‘activation’: ‘relu’, ‘alpha’: 0.0001, ‘hidden_layer_sizes’: (100, 100), ‘learning_rate’: ‘adaptive’, ‘solver’: ‘adam’} Mean cross-validation score: 0.9476 SD cross-validation score: 0.0904

For the MUDC dataset, the best classification algorithm was the neural network MLP classifier algorithm, which achieved 94% accuracy with normalized data and 30 reduced features (Table 6, 7).

**Table 7.** MUDC dataset Classification Accuracy Scores

**Classifier**	**Accuracy**
LR	87%
RF	90%
NN	94%
**Random Forest**	**Classifier results**
Accuracy	94%
F1 Weighted	93% (+/- 0.02)
Mean AUC	93% (+/- 0.02)

### Cross-Language Evaluation

We evaluated the accuracy of the two models trained on the ADRess and MUDC datasets separately by validating them in other languages for independent validation. Using the complete ADRess dataset as the training dataset and the MUDC dataset as the validation dataset, the random forest model achieved the highest accuracy of 72% for the labels and a root mean square error (RMSE) of 6.02 for the MMSE predictions. The Neural Network MLP model trained on the complete MUDC dataset achieved 76% label accuracy when tested on the ADRess dataset for validation. These results indicate the potential power of acoustic features independent of the underlying linguistic properties of the language, such as word order, agglutination, noun cases, and grammatical markers.

### Linguistic Analysis

Linguistic analysis was performed on the ADRess dataset to compare with acoustic features. The ADRess dataset provided transcriptions in CHAT file format for both the test and training datasets, which needed parsing of patient words from the file. For preprocessing, interviewer and redundant words were programmatically removed from these transcriptions. We used Python BERT libraries to extract 20 linguistic features, such as the number of words, number of unique words, speech rate, number of sentences, sentence complexity, and clarity score. Our initial experiments using linguistic features for classification achieved a very low accuracy of 45% with the RF classifier algorithm with normalized data and 30 reduced features (Table 8).

**Table 8.** Linguistic Analysis Accuracy Rates

Algorithm	Optimized Parameters and Accuracy Rates
Data normalized – Feature selection performed – 20 Features	
LR	Best hyperparameters: {‘C’: 1, ‘penalty’: ‘l2’} Mean cross-validation score: 0.2873
RF	Best hyperparameters: {‘max_depth’: 50, ‘max_ features’: ‘sqrt’, ‘n_estimators’: 50} Mean cross-validation score: 0.4591
NN	Best hyperparameters: {‘alpha’: 0.0001, ‘hidden_layer_ sizes’: (100,)} Mean cross-validation score: 0.2873

## Discussion

This study used a publicly available English dataset and an in-house dataset that was collected in a Turkish-speaking dementia clinic. This study is the first machine learning study in the literature presenting a benchmark dataset of audio features from Turkish patients diagnosed with mild cognitive impairment, this study diverges from the predominant literature focusing on English language speakers by conducting research in Turkish. Turkish, classified within the Altaic language group alongside Finnish, Korean, and other Turkic languages, exhibits distinctive phonological therefore sound based properties such as vowel harmony, where vowels within a word tend to coalesce based on shared features such as frontness or rounding. In contrast, Indo-European languages commonly share phonological (sound-based) features, including distinct sounds like the Indo-European laryngeals.

As of 2022, the native speakers of Turkish number approximately 400 million, constituting approximately 5% of the global population (15). Expanding our research of sound-based dementia diagnosis to cover other non-Indo-European languages has the potential to enhance the accuracy of early dementia detection for patients across the linguistically diverse non-English-speaking world.

Furthermore, our study offers an additional benefit: non-English-speaking dementia patients within the Indo-European language group may also derive utility from our findings due to the language-independence of our underlying machine learning model.

This approach enabled us to assess whether audio features alone can be utilized to estimate the course of dementia in different populations independent of the linguistic structure of the language. By conducting the audio recordings firsthand at the clinic immediately after the written MMSE test, some patients testified that audio recordings were more convenient than the written format test, while others found it even harder to recall the word ‘Cookie Jar’, which became frustrated and needed to repeat the recording several times.

The random forest and MLP neural network classification methods yielded high accuracy rates of approximately 94%, showing that acoustic features can be used independent of the linguistic features of the underlying language to create a prediction model. The accuracy rate was 52% before augmentation and feature extraction on the ADRess dataset and 65% on the MUDC dataset. Therefore, feature extraction and augmentation contributed significantly to the accuracy of the models. For comparison, we performed a linguistic analysis on the ADRess dataset for which the transcriptions were available. However, the random forest algorithm’s highest accuracy rate was 45%.

### Limitatons

Even though we increased Turkish dataset from a sample size to 300 by augmentation, it is small compared to the Indo-European dataset. It is limited to a population from a small city in western region with regional language characteristics which might not be representing general Turkish linguistic characteristics. Moreover, Turkish training dataset is limited to mild cognitive delay although Adress dataset contains a wider spectrum of the disease.

## Conclusion

Achieving a high accuracy rate with two different machine learning classifiers in two distinct languages demonstrates the potential of utilizing spontaneous speech (SS) recordings to predict MMSE scores and track the cognitive impairment progress of dementia patients collected at-home by users themselves or their caregivers. Our study highlights the critical importance of the audio features in machine learning models, which can outperform the linguistic features regardless of the language. Our results suggest that adopting a multilingual approach with larger datasets could result in more precise machine learning models. This, in turn, could assist other researchers in software development aimed at monitoring dementia progression more conveniently.
